# Impact of Early Postnatal Maternal Separation Stress on Pancreatic Function in Rodents: A Systematic Review and Meta-Analysis

**DOI:** 10.3390/ijms26209927

**Published:** 2025-10-12

**Authors:** Laura García-Orozco, Josue Rivadeneira, Bélgica Vásquez

**Affiliations:** 1 Doctoral Program in Morphological Sciences, Faculty of Medicine, Universidad de La Frontera, Temuco 4780000, Chile; l.garcia05@ufromail.cl; 2 Center of Excellence in Morphological and Surgical Studies, Universidad de La Frontera, Temuco 4811230, Chile; 3 Zero Biomedical Research, Quito 170403, Ecuador; j.rivadeneira01@ufromail.cl; 4 Doctoral Program in Medical Science, Universidad de La Frontera, Temuco 4811230, Chile; 5 Department of Basic Sciences, Faculty of Medicine, Universidad de La Frontera, Avenida Francisco Salazar 01145, Temuco 4811230, Chile

**Keywords:** maternal separation, early stress, pancreas, insulin resistance

## Abstract

Early postnatal stress is a critical factor in metabolic programming. Maternal separation (MS) in rodents, a widely validated model, has been linked to pancreatic alterations. This systematic review and meta-analysis aimed to evaluate the effect of MS on pancreatic morphology and function in rodents. This review followed the PRISMA and SYRCLE guidelines, with a protocol registered in PROSPERO (CRD420251004633). Experimental studies in rodents comparing MS with standard rearing, which reported pancreatic morphofunctional and metabolic parameters, were included. A comprehensive search was performed in the Web of Science, Embase, Medline, Scopus, BIREME-BVS, and SciELO databases until March 2025, without language restrictions. Extracted data included glucose, insulin, insulin sensitivity indices (QUICKI, HOMA), and glucose tolerance tests (GTTs). Meta-analyses were performed using random-effects models, and subgroup analyses were applied to explore sources of heterogeneity. Of 491 references, 25 studies were included in the meta-analysis, which showed that MS was associated with significantly higher glucose levels (SMD −0.41; 95% CI: −0.71 to −0.11) and worse GTT response (SMD −1.02; 95% CI: −1.23 to −0.82). Furthermore, the QUICKI index was significantly decreased (SMD 0.75; 95% CI: 0.14 to 1.35), indicating insulin resistance. MS in rodents induces pancreatic alterations associated with insulin resistance and glucose intolerance, suggesting that early stress could program long-term metabolic vulnerability.

## 1. Introduction

Chronic stress in childhood, particularly that resulting from adverse childhood experiences (ACEs) such as physical, emotional, and sexual abuse; physical and emotional neglect; and parental separation or divorce, among others [1], is recognized as a determining factor in developmental programming and susceptibility to physical and mental illnesses throughout life [2]. ACEs generate cumulative and potentially synergistic adverse effects, which are particularly detrimental when they occur during critical periods of neurobiological sensitivity and plasticity [3,4,5,6].

Human studies have revealed that the more types of ACEs individuals reported, the greater their risks for unhealthy behaviors (e.g., smoking or sexual risk-taking) and infectious, chronic, and non-communicable diseases [7,8,9]. However, significant ethical limitations of human studies have highlighted the need to use animal models to identify the processes and mechanisms through which early childhood stress impacts development [10]. Added to this are the methodological limitations and possible biases present in observational studies in humans [11], which reinforces the need for preclinical studies that allow exploring these relationships under more controlled conditions. In this context, experimental models using rodents are the most commonly employed today [12].

Among the most widely used experimental paradigms to induce stress in early stages in rodents is maternal separation (MS), which consists of systematically interrupting the mother–offspring interaction during the first two weeks of the postnatal period, a stage characterized by a hyporesponse to stress due to a low activation of the hypothalamic–pituitary–adrenal (HPA) axis in the face of adverse stimuli [13,14,15,16]. This model has primarily enabled the establishment of causal links between early stress and neurobehavioral, cognitive, and emotional alterations [17,18,19].

Complementing neuropsychological findings, recent research has investigated the effects of early-life stress on peripheral organs such as the pancreas. In this context, MS has been identified as inducing significant changes in pancreatic function in rodents, primarily mediated by stress-induced mechanisms that alter plasma insulin levels, β-cell function, oxidative stress, and glucose metabolism [20,21,22]. These alterations contribute to the development of metabolic disorders later in life, highlighting the importance of early developmental conditions in shaping long-term metabolic health and the potential for early interventions to mitigate these effects.

Based on the above, the objective of this review is to evaluate the effect of MS on pancreatic morphology and function in rodents.

## 2. Methods

### 2.1. Protocol and Registration

This systematic review was registered with PROSPERO (CRD420251004633) and conducted following a protocol developed a priori in accordance with the guidelines of the SYRCLE (Systematic Review Centre for Laboratory Animal Experimentation) statement [23]. The manuscript was prepared in accordance with the recommendations of the PRISMA (Preferred Reporting Items for Systematic Reviews and Meta-Analyses) guidelines [24].

### 2.2. Eligibility Criteria

Experimental studies in rodents that compared the effects of MS with a control group (without intervention) on pancreatic morphology and function were eligible for inclusion, with no restrictions on language or publication date.

Systematic reviews, narrative studies, letters to the editor, and other documents that did not provide primary information, as well as studies conducted with genetically modified rodents, were excluded from the review. Furthermore, primary articles that did not report the results of the MS and control groups separately were also excluded.

### 2.3. Information Sources

We conducted searches in the following metasearch engines, libraries, and databases: Web of Science (WoS), Embase, Medline, Scopus, BIREME-BVS, and SciELO. Manual searches were also performed, including cross-referencing of the selected articles and reviewing the gray literature.

### 2.4. Search Strategy

**Figure 1:**
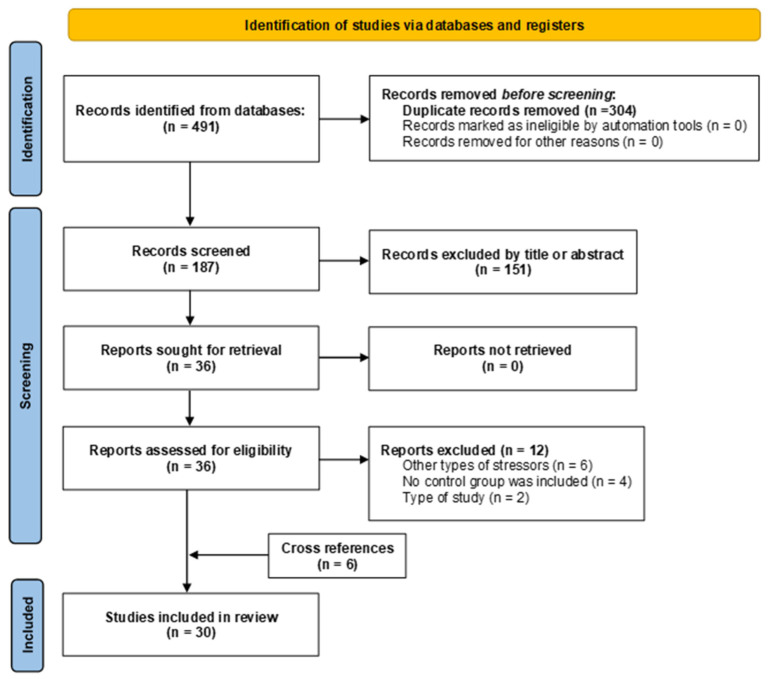
PRISMA flowchart summarizing study selection.

The search was performed using the PICO components (population-based study [P: Rodents], intervention [I: MS], comparator [C: Rodents with a standard rearing model without manipulation], and outcome [O: Assessments of pancreatic functionality or morphology]). Sensitive searches were performed by adapting the search strategy to each information source, considering the use of MeSH, DeCS, and Emtree terms, as well as free terms incorporated through Boolean operators (Supplementary Materials Table S1). The search strategy was independently peer-reviewed according to the Peer Review of Electronic Search Strategies (PRESS) checklist. The search was last updated on 4 March 2025. The study selection process is documented using a PRISMA diagram (Figure 1).

### 2.5. Selection Process

The documents identified from each information source were imported into Rayyan^®^ software (version 1.6.1), which automatically and manually removed duplicates. Two authors (JR and LGO) independently screened the titles and abstracts using the eligibility criteria. The same authors then retrieved the full texts of potentially eligible studies and assessed them independently. The authors resolved any discrepancies by consensus, consulting a third reviewer (BV) if necessary to make the final decision.

### 2.6. Data Collection Process

Data extraction was performed independently by two researchers (JR and LGO). Data were then compiled in a standardized and pre-validated format using Microsoft Excel^®^ (version 16.81).

### 2.7. Data Items

We conducted a comprehensive search, including articles reporting glucose, insulin, and glucagon levels; glucose tolerance tests (GTTs); insulin tolerance tests (ITTs); pancreatic islet isolation and culture (in vitro insulin secretion and insulin content); pancreatic histological and morphological studies; and metabolic assessments using insulin function and sensitivity indices such as HOMA (Homeostasis Model Assessment), HOMA-B (Homeostasis Model Assessment of Beta-cell function), ISI (Insulin Sensitivity Index), and QUICKI (Quantitative Insulin Sensitivity Check Index).

Additionally, various markers related to redox status were analyzed as outcomes; these included antioxidant enzymes such as catalase (CAT), superoxide dismutase (SOD), glutathione peroxidase (GPx), glutathione reductase (GR), and glutathione S-transferase (GST), along with ferric-reducing ability potential (FRAP), an indicator of total antioxidant capacity. Reduced glutathione (GSH) was also assessed. Malondialdehyde (MDA) served as a marker of oxidative damage, while nitric oxide synthase (NOS), interleukin-1β (IL-1β), and insulin-like growth factor 1 (IGF-1) were included due to their roles in inflammation and metabolic regulation.

### 2.8. Study Risk of Bias Assessment

Two investigators (JR and LGO) independently performed the Risk of Bias (RoB) assessment using the SYRCLE RoB tool for animal studies [25]. This tool contains 10 items addressing six types of domains: selection bias (sequence generation, baseline characteristics, and allocation concealment), performance bias (random housing and blinding of interventions), detection bias (random outcome assessment and blinding of outcome assessment), attrition bias (incomplete outcome data), reporting bias (selective outcome reporting), and other biases. Each entry was assessed based on the corresponding flagging questions: a “Yes” answer reflected a low RoB, “No” corresponded to a high RoB, and “Unclear” indicated an unclear RoB due to insufficient information to make a definitive judgment. Answering “No” to any of the relevant flagging questions indicated a high RoB for that specific domain.

### 2.9. Synthesis Methods

We conducted a qualitative and descriptive analysis of the evidence. Statistical analyses were conducted in R version 4.4.2 using the “metafor” package. We performed random-effects meta-analyses on outcomes with two or more articles using the restricted maximum likelihood (REML) method, using the standardized mean difference and its standard deviation to calculate the standardized effect size for each outcome.

When necessary, we converted data to ensure comparability. Studies lacking derivable summary statistics were excluded from the quantitative synthesis but included in the qualitative summary. In subgroup analyses, the total number of studies may not match the overall count, as some studies contributed data to more than one category (e.g., sex or age). In contrast, others did not report all variables.

### 2.10. Assessment of Heterogeneity

Chi^2^, Tau^2^, and I^2^ statistics were used. Subgroup analyses were performed to study heterogeneity, considering the following characteristics: species, sex, age at the time of testing, and duration of the MS.

### 2.11. Assessment of Reporting Bias

We examined funnel plot asymmetry and applied Egger’s test only to meta-analyses that included at least 10 studies, following the recommendations of the Cochrane Handbook for Systematic Reviews of Interventions [26,27]. We set this threshold because, with fewer than 10 primary studies, these tests have low statistical power and their results are considered unreliable for detecting publication bias or small-study effects.

## 3. Results

### 3.1. Study Selection

A total of 491 studies were identified. Of these, 304 were excluded because they were duplicate records. The remaining 187 articles underwent title and abstract screening, resulting in the exclusion of 151 articles that did not meet the inclusion criteria. Of the 36 articles reviewed in full, 12 were excluded for not meeting the eligibility criteria. Additionally, 6 studies were identified through cross-referencing, resulting in 30 studies included in the final analysis [20,21,22,28,29,30,31,32,33,34,35,36,37,38,39,40,41,42,43,44,45,46,47,48,49,50,51,52,53,54].

### 3.2. Study Characteristics

The studies included in the analysis, along with their general characteristics, are presented in Table 1. Of the eligible studies, 76.67% were conducted in rats (n = 23) and 23.33% in mice (n = 7). Half of the investigations used only male animals (n = 15), 40% included both sexes (n = 12), and 10% used females (n = 3). The age at which pancreatic morphofunctional evaluations were performed was grouped into six categories: (1) infant (0–21 PND), (2) adolescent (22–59 PND), (3) young adult (60–120 PND), (4) adult (121–300 PND), (5) aged (>300 PND), and (6) not reported. Accordingly, 43.33% of the studies performed measurements in young adult rodents (n = 13), 33.33% in adults (n = 10), 16.67% in adolescents (n = 5), 13.33% in infants (n = 4), 6.67% in aged rodents (n = 2), and 3.33% did not report the age (n = 1).

The characteristics of the various MS protocols used in the included studies are presented in Table 2. In 46.67% of cases, the postnatal day of initiation of the protocol was day 2 (n = 14), 43.33% of the studies started on the first postnatal day (n = 13), 6.67% on day 9 (n = 2), and 3.33% on postnatal day 8 (n = 1). Regarding the daily duration of MS, the studies were classified into three categories: brief (≤30 min), moderate (31 min to 3 h), and prolonged (>3 h). Most studies (76.67%, n = 23) applied protocols with moderate duration, followed by those that used prolonged separations (23.33%, n = 7) and, to a lesser extent, brief ones (3.33%, n = 1). Regarding the total duration of the MS protocol, studies were classified into four categories: ≤1 day, 2–7 days, 8–14 days, and 15–21 days. The highest proportion of studies (43.33%, n = 13) applied the protocol for 15–21 days, followed by 8–14 days (40%, n = 12), ≤1 day (10%, n = 3), and 2–7 days (6.67%, n = 2).

Regarding the conditions under which the MS was applied, the categorization of this variable showed two main aspects: whether the separation was carried out as a group or individually, and whether the mother remained in the same room as the pups or in a different one. Based on these criteria, six categories were established: (1) group separation with the mother in the same room, (2) group separation with the mother in another room, (3) group separation without details about the mother’s location, (4) individual separation with the mother in another room, (5) individual separation without details about the mother’s location, and (6) no information reported. Thus, the most frequent category was group separation without details about the mother’s location, used in 43.33% of the studies (n = 13), followed by group separation with the mother in another room in 20% (n = 6). Other conditions included group separation with the mother in the same room (13.33%, n = 4), individual without details about the mother’s location (10%, n = 3), individual with the mother in another room (6.67%, n = 2), and studies that did not report this information (6.67%, n = 2).

### 3.3. Risk of Bias in Studies

**Figure 2:**
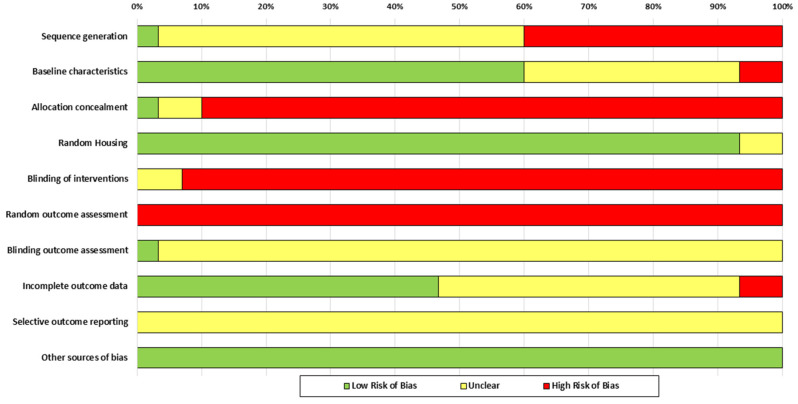
Risk of bias assessment for the 30 included studies, as per the SYRCLE RoB tool. Low risk of bias is shown in green, unclear risk in yellow, and high risk of bias in red.

The results of the RoB assessment are summarized in Table 3 and Figure 2. Random sequence generation presented unclear risk in more than half of the studies (57%) and high risk in 40%, due to a lack of details on the randomization procedure. Allocation concealment was one of the most compromised aspects, with 90% of studies classified as high risk, due to not describing any methods or performing the procedure with prior knowledge. Regarding blinding of interventions, 93% of the studies showed a high risk, since this aspect was not reported.

Randomization in outcome assessment was not applied in any of the studies, implying a high risk in all cases. Meanwhile, assessor blinding was predominantly judged as unclear due to insufficient information, although it was considered unlikely to affect the results. Regarding baseline characteristics, 60% of the studies presented low risk, while the remaining studies showed unclear or high risk due to limited details on post-intervention conditions.

### 3.4. Results of Individual Studies

We conducted the meta-analysis on 25 studies previously included in the systematic review. The analysis focused on five metabolic parameters: glucose, insulin, QUICKI and HOMA indices, and GTTs. Although some studies addressed redox status and molecular mediators, we did not include these outcomes in the meta-analysis due to the limited availability of comparable data across studies. Reported redox assessments included antioxidant enzymes such as CAT in 6.67% of the studies (n = 2), SOD, GPx, GR, and GST, each in 3.33% (n = 1). Antioxidant capacity indicators, such as FRAP and GSH (3.33%, n = 1 each), and oxidative damage markers, such as MDA (6.67%, n = 2), were also measured. Finally, some studies evaluate specific molecular mediators, NOS, IL-1β, and IGF-1, each reported in 3.33% of studies (n = 1).

#### 3.4.1. Glucose

**Figure 3:**
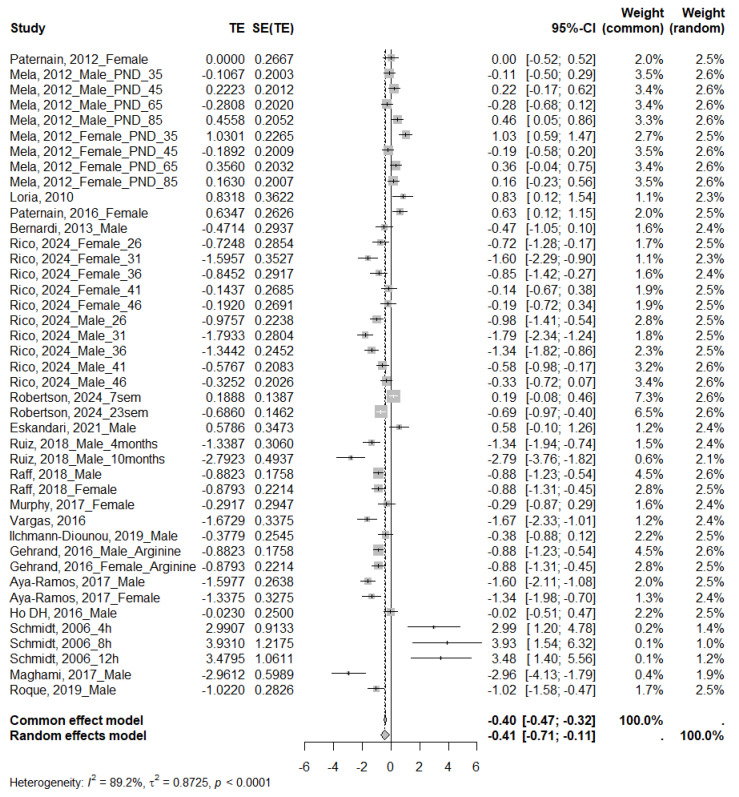
Forest plot showing the effect size of glucose. TE: Treatment Effect; SE(TE): Standard Error of the Treatment Effect; Significant differences (*p* < 0.05) [21,28,30,34,35,36,37,39,40,41,42,43,44,45,46,47,49,53,54].

**Figure 4:**
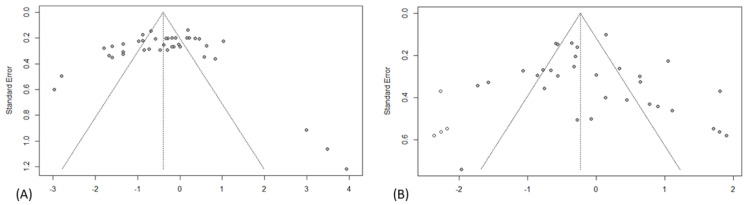
Funnel plots indicating publication bias of included studies: (**A**) Glucose; (**B**) Insulin.

Twenty-three studies analyzed glucose levels, of which 19 were eligible for the meta-analysis. The analyses showed a significant effect, with higher glucose levels in the MS-exposed group (SMD −0.41; 95% CI: −0.71 to −0.11) (Figure 3). Inter-study heterogeneity was high (I^2^ = 89.2%, τ^2^ = 0.8725, *p* < 0.0001), as demonstrated by performing subgroup analyses (Table 4 and Supplementary Materials Figure S1). A significant increase in glucose was evident in rats (SMD = −0.59; 95% CI: −0.87 to −0.30), males (SMD = −0.77; 95% CI: −1.13 to −0.34), and adolescents (SMD = −0.74; 95% CI: −1.13 to −0.34). For MS only at moderate separation times (SMD = −0.67; 95% CI: −1.10 to −0.24). Furthermore, the test for subgroup differences for species and age at testing was statistically significant (*p* = 0.0206 and *p* < 0.0001, respectively), suggesting that these variables might influence the magnitude of the observed effect. Visual inspection of the funnel plot (Figure 4) indicated no apparent asymmetry, which Egger’s regression test confirmed (*p* = 0.7453).

#### 3.4.2. Insulin

**Figure 5:**
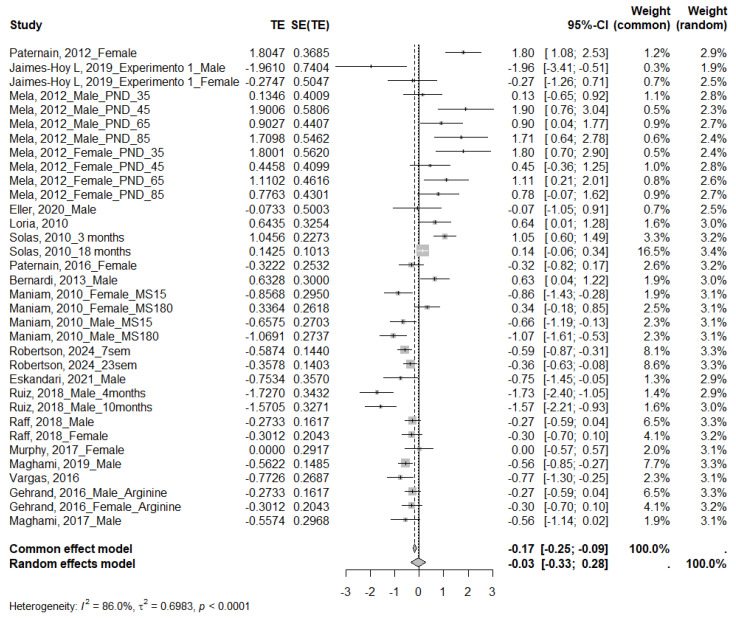
Forest plot showing the effect size of insulin. TE: Treatment Effect; SE(TE): Standard Error of the Treatment Effect; Significant differences (*p* < 0.05) [20,21,30,31,32,34,35,36,37,40,41,43,44,45,46,48,50,54].

Twenty-two studies measured insulin levels, of which eighteen were eligible for the meta-analysis. The MS group showed an upward trend; however, this difference was not statistically significant (SMD = −0.03; 95% CI: −0.33 to 0.28) (Figure 5). Given the high heterogeneity across studies (I^2^ = 86.0%, τ^2^ = 0.6983, *p* < 0.0001), subgroup analyses were also performed (Table 5 and Supplementary Materials Figure S2). A significant decrease in insulin levels was identified in the MS group for prolonged separations (SMD = 1.01; 95% CI: 0.56 to 1.45) and an increase for short separations (SMD = −0.75; 95% CI: −1.14 to −0.36), as well as for studies conducted in mice (SMD = −0.45; 95% CI: −0.66 to −0.24). The test for differences between subgroups for species, sex, and duration of MS was statistically significant (*p* = 0.0224, *p* = 0.0381, and *p* < 0.0001, respectively), suggesting that these variables may influence the magnitude of the observed effect. Visual inspection of the funnel plot (Figure 4) indicated no apparent asymmetry, which Egger’s regression test confirmed (*p* = 0.2486).

#### 3.4.3. QUICKI

**Figure 6:**
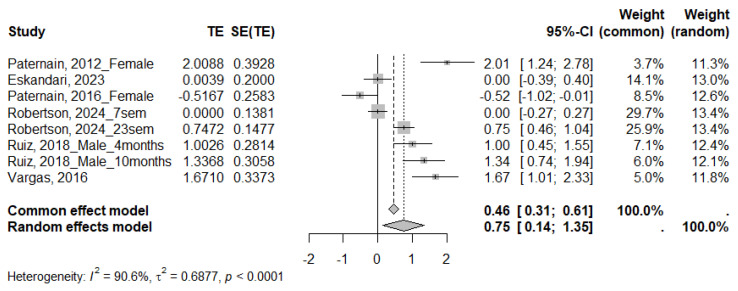
Forest plot showing the effect size of QUICKI index. TE: Treatment Effect; SE(TE): Standard Error of the Treatment Effect; Significant differences (*p* < 0.05) [22,35,40,41,46,54].

Six studies reported the QUICKI index, all of which were included in the meta-analysis, which showed a significant decrease in this parameter in animals subjected to MS (SMD 0.75; 95% CI: 0.14 to 1.35; *p* < 0.0001) (Figure 6). Given the high heterogeneity across studies (I^2^ = 90.6%, τ^2^ = 0.6877, *p* < 0.0001), subgroup analyses were also performed (Table 6 and Supplementary Materials Figure S3). The meta-analysis revealed a significant decrease in the QUICKI index only in rats (SMD = 0.89; 95% CI: 0.10 to 1.68) and in males (SMD = 1.16; 95% CI: 0.75 to 1.56). When analyzing the duration of the MS, moderate-duration protocols showed a significant effect (SMD = 0.75; 95% CI: 0.14 to 1.35). The subgroup differences test showed no significant differences.

#### 3.4.4. HOMA

**Figure 7:**
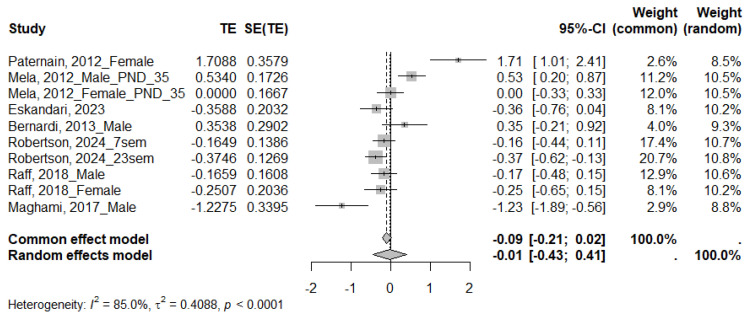
Forest plot showing the effect size of HOMA index. TE: Treatment Effect; SE(TE): Standard Error of the Treatment Effect; Significant differences (*p* < 0.05) [22,34,35,36,43,45,54].

For the HOMA index, reported in eight studies, seven met the criteria for inclusion in the meta-analysis. The MS group showed an upward trend; However, this difference was not statistically significant (SMD = −0.01; 95% CI: −0.43 to 0.41) (Figure 7). Given the high heterogeneity across studies (I^2^ = 85.0%, τ^2^ = 0.4088, *p* < 0.0001), subgroup analysis was also performed (Table 7 and Supplementary Figure S4). The HOMA index increased in mice (SMD = −0.28; 95% CI: −0.48 to −0.07). No statistically significant differences were found for the other variables analyzed. The subgroup differences test showed no significant differences.

#### 3.4.5. GTTs

**Figure 8:**
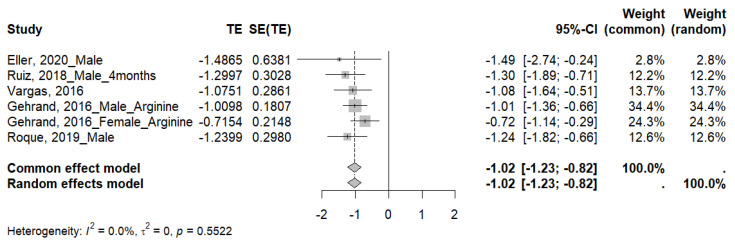
Forest plot showing the effect size of GTTs. TE: Treatment Effect; SE(TE): Standard Error of the Treatment Effect; Significant differences (*p* < 0.05) [37,41,46,49,50].

Of eight studies that performed the GTTs, five met the criteria for inclusion in the meta-analysis, showing significantly higher values in rodents exposed to MS (SMD −1.02; 95% CI: −1.23 to −0.82) (Figure 8). Although no heterogeneity was evident among the studies analyzed (I^2^ = 0%, τ^2^ = 0, *p* = 0.5522), we conducted a subgroup analysis (Supplementary Materials Figure S5) to explore possible biological or methodological factors that could affect the magnitude of the effect. Table 8 provides detailed information on these analyses. The subgroup analysis revealed significant effects in all categories analyzed, including species, sex, and developmental stage, with no statistically significant differences between subgroups.

## 4. Discussion

In this systematic review and meta-analysis, we sought to analyze the effects of MS as a model of early postnatal stress on pancreatic morphology and function. To our knowledge, this is the first meta-analytic investigation of these outcomes in rodents. The evidence gathered indicated that MS is associated with metabolic alterations, particularly a significant increase in glucose levels and response to GTTs, as well as a marked decrease in the QUICKI index, suggesting possible alterations in insulin sensitivity. Subgroup analyses identified potential biological and methodological moderators, such as species, sex, age at assessment, and duration of the MS protocol, which could explain some of the variability observed in the results. Therefore, consideration of these variables should be consistent with the rationale and objectives of the experimental design.

### 4.1. Glucose

Glucose levels are a relevant metabolic marker due to their central role in energy production and their usefulness in assessing the overall functional status of the organism. Previous studies have shown that early exposure to stress, such as MS, can interfere with the functional maturation of the HPA axis and alter metabolic programming, affecting key organs such as the liver, adipose tissue, and pancreas [42,54,55,56]. Consistent with this background, the results of this review showed a significant increase in glucose levels in animals subjected to MS. This sustained elevation can trigger multiple adverse consequences, including oxidative stress, insulin resistance, glucose intolerance, and metabolic and histopathological alterations, among others [57,58,59].

Subgroup analyses revealed that species and age at testing contribute to the heterogeneity observed across studies, with greater susceptibility evident in rats and in animals evaluated during adolescence. In this regard, early MS stress differentially affects rats and mice due to species-specific characteristics. In rats, the standard 3 h of MS are sufficient to induce significant metabolic alterations [43,46]. However, in mice, this same period often triggers an increase in maternal care, which could attenuate the effects of separation [60]. Therefore, in this species, prolonged periods of MS (e.g., 8 h) are required to induce alterations in peripheral metabolic markers such as glucose, leptin, and ghrelin [28].

The above may help interpret several of the findings in this review. First, 79% of the studies involved rats, and only a minority used mice, sometimes without adequately considering the differences in the separation times required for each species [47,50,54]. While subgroup analyses based on MS duration did not reveal a clear impact on overall heterogeneity, it is worth noting that only moderate separation times were associated with significant alterations in glucose levels. This effect has been predominantly reported in rats. These results reinforce the idea that both the species used and the duration of the protocol are key variables in the expression of metabolic alterations associated with MS.

In addition, another modulating factor identified was the developmental stage at which the animals were evaluated. In this sense, the observation of significant effects only in rodents studied during adolescence may be explained by the immaturity of the HPA axis, the brain maturation process, and several neurobiological changes characteristic of this stage, reflected in a prolonged corticosterone response to stress. This sustained activation of the HPA axis can amplify the effects of stress, making them more significant and lasting during adolescence compared to other developmental stages [61,62].

### 4.2. Insulin

Insulin and glucose levels are closely related, as the latter plays a fundamental role in tissue glucose uptake and storage. In this context, the findings of the present meta-analysis suggest that, while glucose levels increase significantly in rodents exposed to MS, insulin levels did not show significant overall differences compared to controls. Peripheral insulin resistance likely accounts for this pattern, in which the pancreas maintains its secretory capacity, but tissues respond less effectively to its action, thus reducing the efficiency of glycemic control [20,43]. In line with this interpretation, previous studies have reported that, after exposure to MS, the insulin content in pancreatic islets may decrease without implying an alteration in its secretion [20,22], which suggests a compensatory response of the pancreas to stress.

Subgroup analyses revealed divergent effects depending on the specific characteristics of the protocol. In particular, a significant increase in insulin was observed in studies with mild separations, whereas studies with severe separations showed a decrease; however, these findings should be interpreted with caution, as only one study evaluated mild separations and another evaluated severe separations. The limited representation precludes firm conclusions and highlights the need for further research to systematically explore how different durations of MS might impact insulin regulation.

In addition to the factors previously discussed, the analyses revealed relevant differences based on sex. Although individual studies did not find significant effects in men or women, the test for differences between subgroups showed statistical significance, suggesting that sex acts as a moderator contributing to the variability of the observed results. This pattern may result from opposing effects on insulin levels in men and women: a trend toward decreased levels in women and increased levels in men. Consequently, no individual comparison reaches statistical significance, although a significant difference is evident between the two groups. These differences may reflect sex-specific endocrine responses to stress, highlighting the need for studies specifically designed to investigate these interactions. These findings are particularly relevant given that the overall effect of maternal separation on insulin levels was not significant.

### 4.3. QUICKI

The QUICKI index, widely used to estimate insulin sensitivity, showed a significant decrease in rodents subjected to MS, suggesting a pattern consistent with insulin resistance. Although the number of studies was limited, subgroup analysis revealed that this effect occurred only in rats and male animals, as well as in those exposed to moderate separation protocols. The fact that the studies reported significant effects only in rats and with moderate separation times is not surprising, given that most of the included studies were conducted in this species and under 3 h deprivation protocols, as previously discussed. Furthermore, it is relevant to consider that all studies evaluating this index used exclusively moderate separation times, which prevented comparisons between different durations. Consequently, we cannot determine whether other durations of MS might produce similar or divergent effects on insulin sensitivity.

Regarding sex, the presence of a significant effect only in men could indicate a greater susceptibility of this group to MS-induced alterations in insulin sensitivity, which is consistent with findings observed on glucose levels in previous studies, where a significant increase was also evident only in men [33,37]. Since the QUICKI score is derived from fasting glucose and insulin levels, an increase in glucose (as observed in men) may directly contribute to a decrease in the score. This greater vulnerability may be due to differentiated physiological responses between sexes, especially in HPA axis activity, hormonal profiles, and metabolic adaptations to stress. Previous research [49] reports that MS leads to increased HPA axis activation in males, resulting in heightened stress responses and higher circulating levels of corticosterone [22], a hormone that can impair glucose tolerance and elevate blood glucose concentrations. In this context, the effects observed on the QUICKI index may represent an indirect manifestation of these heightened endocrine responses, highlighting the importance of considering sex as a moderating variable when assessing the metabolic consequences of early postnatal stress.

### 4.4. GTTs

Regarding the GTTs, the results of the meta-analysis showed a significant overall effect of MS, indicating an alteration in the animals’ ability to handle glucose overload. This pattern suggests a decrease in the effectiveness of the glycemic regulatory system, probably associated with a state of insulin resistance. Under normal physiological conditions, after glucose administration, a transient increase in insulin levels and rapid glucose uptake by peripheral tissues are expected. However, in animals subjected to MS, this response appears to be compromised, reflected in a larger area under the curve in the GTTs [37,41,46,47,49,50]. This functional alteration is consistent with the changes observed in basal glucose and insulin levels, as well as in the QUICKI index. These findings reinforce the hypothesis of metabolic dysfunction induced by early stress.

Subgroup analyses revealed significant effects across all evaluated categories (species, sex, age, and duration of MS), suggesting that the studied intervention may have a robust and generalizable impact on glucose responses to an exogenous glucose load. However, this finding relies on a limited number of studies (n = 5), all of which used moderate-duration MS protocols, preventing direct comparisons of different early-life stress regimens on this functional test.

Another key limitation was the low use of mice as an experimental model, even though this species exhibits a metabolic physiology that is more comparable to humans [63,64] than that of other frequently used models, such as rats. This underrepresentation may stem from the fact that many of the included studies did not primarily aim to evaluate metabolic parameters, but focused instead on behavioral, neuroendocrine, or developmental outcomes. However, this methodological limitation may constrain the generalization of some findings to human contexts. Therefore, promoting preclinical research that incorporates mice as an experimental model could enhance the translational relevance of the results and deepen our understanding of the metabolic mechanisms underlying early-life stress.

On the other hand, the concomitant observation of impaired insulin sensitivity (QUICKI) and increased glucose intolerance (also supported by GTT results in the included studies) raises a relevant translational question. The clinical literature reports comparable associations in humans exposed to adverse childhood experiences, where such experiences have been identified as an independent risk factor for the development of prediabetes, characterized by glucose intolerance, reduced insulin sensitivity, and altered beta-cell function [65,66]. Consistently, other reports indicate that psychological sequelae resulting from childhood maltreatment represent a critical pathway for the development of endocrine diseases, particularly type 2 diabetes and metabolic disorders linked with HPA axis dysregulation [67]. Establishing this parallel suggests that the metabolic pathways observed in rodents undergoing MS may reflect pathophysiological mechanisms relevant to humans, thereby reinforcing the clinical value of these findings and underscoring the need for comprehensive research that connects preclinical data to humans.

### 4.5. HOMA

Regarding the HOMA index, the results of the meta-analysis did not show a significant overall effect of MS on insulin resistance. This index, along with QUICKI, is one of the most widely used methods for assessing insulin sensitivity based on fasting glucose and insulin measurements [68]. Although both share the same variables, they differ in the way they are processed mathematically, since QUICKI applies a logarithmic transformation that improves its sensitivity. In this sense, studies that have performed calibration analyses indicate that QUICKI tends to have better predictive accuracy, as well as better reproducibility, compared to HOMA in rodent models, although both indices are less predictive in rodents than in humans due to intrinsic physiological differences [68,69,70]. Taken together, the above could suggest that, under the conditions evaluated, MS does not generate consistent changes in the HOMA index, which does not necessarily indicate the absence of metabolic dysfunction, but rather highlights the need to consider multiple complementary indicators for a more accurate assessment of insulin status.

Subgroup analyses revealed no statistically significant differences between the moderators assessed, suggesting that other unaccounted biological or methodological variables could contribute to the high level of heterogeneity observed. This variability could reflect complex interactions between experimental factors, making it difficult to identify consistent effects using this index and highlighting the need for more standardized methodological approaches in future studies.

Finally, we consider it crucial to highlight that, while most of the studies included in this review do not specifically seek to evaluate pancreatic metabolism or function, researchers include measurements of parameters such as fasting glucose and insulin as part of the general characterization of the animals’ physiological status. However, these isolated measurements, although informative, are insufficient to fully understand the mechanisms through which early stress can alter pancreatic function. Some studies have examined more specific aspects, including pancreatic islet composition, insulin content, and ex vivo insulin secretion [20,22,37], but such research remains scarce and focuses on a limited number of experimental models. Therefore, researchers need to promote studies that incorporate comprehensive assessments of the endocrine pancreas, including morphological, functional, and molecular analyses, which will allow for clearer links between MS and changes in glycemic regulation.

Furthermore, this review did not identify studies that specifically addressed the potential effects of MS on the exocrine pancreas, despite its relevance to digestive function. Recently, a study [52] reported that early-stage stress can modify the activity of antioxidant enzymes in the pancreas, suggesting a possible role of oxidative stress as a mechanism underlying pancreatic alterations. However, these findings are still incipient and require further investigation through systematic studies that consider both the exocrine and endocrine functions of the pancreas to achieve a more complete understanding of the metabolic consequences of MS.

### 4.6. Limitations

One of the main limitations of this systematic review and meta-analysis was the high proportion of studies with a moderate or high risk of bias, particularly in areas such as random sequence generation, allocation concealment, and blinding of interventions and assessors, which could have influenced the internal validity of the results. This issue is widely recognized in the preclinical field, underscoring the need to adopt standardized reporting practices. Initiatives such as the ARRIVE [71] guidelines and the promotion of the SYRCLE RoB [25] tool seek to provide a framework for more transparent reporting in animal research, helping to minimize bias and improve reproducibility. We believe that greater adherence to these recommendations will be crucial to strengthening the translational value of preclinical studies and improving the robustness of future systematic reviews and meta-analyses in this field.

On the other hand, although we included 30 studies, not all of the included studies provided complete data for every metabolic parameter analyzed. This limitation reduced the statistical power of some meta-analyses, particularly those assessing the QUICKI and HOMA indices and the GTTs. In addition, several subgroup analyses relied on a small number of included studies. This methodological constraint decreases the precision of the pooled estimates and increases uncertainty in the results. Therefore, we advise interpreting the findings of these subgroups with caution, recognizing that both the magnitude and direction of the effects may be influenced by the limited representation of included studies and the inherent variability of the experimental protocols.

Considerable methodological heterogeneity was also evident among the included studies, particularly in relation to the biological characteristics of the animals (species, sex, and age at the time of evaluation) as well as the total duration of the MS protocol. These variations between studies make direct comparison of results difficult and may have contributed to the high statistical heterogeneity observed in several of the quantitative analyses performed.

Another relevant limitation was the predominantly partial approach of the metabolic assessments. Most studies focused on isolated measurements of glucose and insulin, without examining other functional or structural aspects of the endocrine pancreas. In addition, we did not identify any studies that directly assessed exocrine pancreatic function. This limitation restricts a comprehensive understanding of the impact of MS on this key organ in metabolic regulation. Finally, although some studies reported alterations in parameters related to redox status and molecular signaling, the small number of studies that examined these outcomes prevented their inclusion in the meta-analysis, which also restricts the conclusions regarding the underlying mechanisms.

## 5. Conclusions

The results of this systematic review and meta-analysis indicate that MS, as a model of early postnatal stress, induces relevant changes in glycemic homeostasis in rodents, particularly evidenced by an increase in glucose levels, a decrease in the QUICKI index, and an altered response in the GTTs. These findings suggest a possible dysfunction in insulin sensitivity induced by early exposure to stress. Although insulin levels and the HOMA index did not show significant overall effects, subgroup analyses revealed differentiated patterns according to species, sex, protocol duration, and developmental stage at the time of testing, highlighting the importance of considering these moderators in the design and interpretation of experimental studies.

Although most researchers have focused their studies on rats, it is essential to promote the use of mice as an experimental model, since mice share greater genetic and physiological similarities with humans in terms of metabolism, which could improve the extrapolation of findings to human health. Furthermore, researchers need to move toward studies that evaluate the pancreas comprehensively, incorporating morphological, functional, and molecular analyses, as this review reveals a notable lack of research that thoroughly examines both endocrine and, to a greater extent, exocrine function, whose role in alterations induced by early stress researchers have not yet systematically investigated.

## Figures and Tables

**Table 1:** Descriptive characteristics and main findings of the included studies.

Authors	Species/ Strain	Total Number (per Group)	Sex	Age of Assessment (PND)	Fasting Before the Tests	Variables Evaluated	Main Findings
Schmidt et al. [28]	Mice/CD1	MS: 8–10, Ctl: 8–10	M/F	8	Yes (4, 8 o 12 h)	Plasma glucose	[↓] Glucose in the MS group at 4, 8 and 12 h vs. Ctl
George et al. [29]	Mice/C57BL/6J	NR	M	10–17	NR	Serum glucose	MS did not produce significant changes
Loria et al. [30]	Rats/Wistar Kyoto	MS: 9–12, Ctl: 9–12	M	84	No	Plasma glucose and insulin	MS did not produce significant changes
Maniam & Morris [31]	Rats/Sprague-Dawley	MS: 11–13, Ctl: 11–13	M/F	133	NR	Plasma insulin	MS 15 min [↑] insulin in F of the MS vs. Ctl, while M showed no statistically significant changes. Prolonged MS (180 min) did not produce statistically significant changes in either M or F
Solas et al. [32]	Rats/Wistar	MS: 28, Ctl: 32	M	84–504	Yes	Plasma insulin	[↓] insulin in the MS group vs. Ctl
Viveros et al. [33]	Rats/Wistar	MS: 12–16, Ctl: 12	M/F	9–10	NR	Circulating glucose	MS 12 h: [↑] glucose in F vs. Ctl0; [↓] in both sexes vs. Ctl12. MS24h and MS36: no change vs. Ctl0, but [↓] vs. Ctl24/36. Ctl36 showed [↑] glucose vs. Ctl0
Mela et al. [34]	Rats/Wistar	MS: 12, Ctl: 12	M/F	102	Yes (12 h)	Plasma glucose and insulin, HOMA	In F with MS: [↓] glucose (PND 35), [↓] insulin (PND 35, 45, 65, 85). In M with MS: [↓] insulin (PND 45, 85); no relevant changes in glucose. HOMA, no significant changes
Paternain et al. [35]	Rats/Wistar	MS: 10, Ctl: 6	F	95	NR	Circulating glucose, serum insulin, HOMA, HOMA-B, QUICKI	[↓] Insulin, HOMA, HOMA-B and, QUICKI in the MS group vs. Ctl. No differences in glucose levels
Bernardi et al. [36]	Rats/Wistar	MS: 7, Ctl: 7	M	126	Yes (6 h)	Plasma glucose, serum insulin, HOMA	MS did not produce significant changes
Gehrand et al. [37]	Rats/Sprague-Dawley	MS: 22, Ctl: 23	M/F	105–133	NR	Plasma glucose, insulin, and glucagon, GTTs, insulin and glucagon, beta cell count	In M and F [↑] glucose. [↑] AUC in M for insulin and glucose, and not in F. No differences in insulin levels, number of β cells, and AUC for glucagon. In M, [↓] glucagon in the MS group vs. Ctl, no differences in F
Ghosh et al. [38]	Rats/Sprague-Dawley	MS: 10, Ctl: 10	M	112–224	Yes	Serum glucose, GTTs, and peritoneal insulin	MS did not produce significant changes
Ho et al. [39]	Mice/C57BL/6J	MS: 5–7, Ctl: 5–7	M	84	No	Plasma glucose	MS did not produce significant changes
Paternain et al. [40]	Rats/Wistar	MS: 8, Ctl: 8	F	182	NR	Serum glucose, plasma insulin, QUICKI	MS did not produce significant changes
Vargas et al. [41]	Rats/Sprague-Dawley	MS: 8–12, Ctl: 8–12	M	67	Yes	Plasma glucose and insulin, QUICKI, GTTs	Glucose, insulin, and QUICKI levels were unchanged. Blood glucose levels in the GTTs were [↑] at 30 and 60 min after injection in the MS group vs. Ctl
Aya-Ramos et al. [42]	Rats/Wistar	MS: 7–10, Ctl: 8–10	M/F	26	NR	Circulating glucose	[↑] Glucose in both M and F in the MS group vs. Ctl
Maghami et al. [43]	Rats/Wistar	MS: 7, Ctl: 7	M	53	NR	Plasma glucose and insulin, HOMA	[↑] Glucose in the MS group vs. Ctl. No differences in insulin levels. [↑] HOMA in the MS group vs. the Ctl
Murphy et al. [44]	Rats/Wistar Kyoto	MS: 6–8, Ctl: 6–8	F	105	Yes (16 h)	Circulating glucose and plasma insulin	MS did not produce significant changes
Raff et al. [45]	Rats/Sprague-Dawley	MS: 22, Ctl: 23	M/F	NR	Yes (Night)	Plasma glucose and insulin, HOMA	No differences in glucose and insulin levels. [↑] HOMA in both M and F in the MS group vs. Ctl
Ruiz et al. [46]	Rats/Sprague-Dawley	MS: 7–10, Ctl: 7–10	M	120–300	Yes (Night)	Plasma glucose and insulin, QUICKI and GTTs	[↑] Glucose and insulin at both 120 PND and 300 PND in the MS group vs. Ctl. [↓] QUICKI at both 120 PND and 300 PND in the MS group vs. Ctl. [↑] AUC in the MS group 120 PND vs. Ctl, unchanged by 300 PND
Ilchmann-Diounou et al. [47]	Mice/C3H/HeN	MS: 8, Ctl: 8	M	350	Yes (6 o 12 h)	Circulating glucose, plasma insulin, GTTs, intraperitoneal insulin tolerance	[↑] Glucose in the MS group vs. Ctl. No differences in insulin levels. The AUC for glucose and insulin [↑] in the MS group vs. the Ctl
Jaimes-Hoy et al. [48]	Rats/Wistar	MS: 4, Ctl: 4	M/F	90	No	Serum insulin	[↑] Insulin in M in the MS group vs. Ctl. No changes are evident in F
Maghami et al. [20]	Rats/Wistar	MS: 34, Ctl: 34	M	53	Yes (16 h)	Plasma insulin, islet isolation	There were no differences in insulin levels or insulin secretion in isolated islets. Islet insulin content showed a significant reduction in the MS group vs. Ctl
Roque et al. [49]	Rats/Sprague-Dawley	MS: 8, Ctl: 8	M	126	Yes (Night)	Circulating glucose, GTTs	[↑] GTTs in MS group vs. Ctl. Glucose without significant changes
Eller et al. [50]	Mice/C57BL/6	MS: 4, Ctl: 4	M/F	189	Yes (6 h)	Serum insulin, GTTs	No differences in insulin levels. [↑] AUC in the MS group vs. Ctl
Eller et al. [51]	Mice/C57BL/6	MS: 6–16, Ctl: 6–16	M/F	126–133	Yes (6 h)	Serum insulin, HOMA, GTTs	MS did not produce significant changes
Eskandari et al. [21]	Rats/Wistar	MS: 7, Ctl: 7	M	72	Yes (16–18 h)	Plasma glucose and insulin, HOMA2-IR, GSH, MDA, IL-1β	[↓] CAT and [↑] MDA in the MS group vs. Ctl. The other variables without significant changes
Eskandari et al. [22]	Rats/Wistar	MS: 10, Ctl: 10	M	70	Yes (16–18 h)	Plasma insulin, HOMA, HOMA-B, QUICKI, ISI, GTTs, islet area, beta cell number, islet isolation, CRFR1	MS only produced changes in the percentage of beta cells, where there was a decrease in the MS group vs. Ctl
Fenton-Navarro et al. [52]	Rats/Sprague-Dawley	MS: 4–5, Ctl: 4–5	M/F	15	NR	CAT, MDA, SOD, GPX, GR, GST, NOS, FRAP	[↑] CAT, MDA in F and not in M, [↑] SOD, GST in both F and M, [↓] GPX, FRAP in both F and M, [↑] GR in M and not in F, [↑] NOS in F and not in M in the MS group vs. Ctl
Rico et al. [53]	Rats/Wistar	MS: 8–10, Ctl: 8–10	M/F	26	No	Circulating glucose	MS did not produce significant changes
Robertson et al. [54]	Mice/C57BL/6J	MS: 14, Ctl: 15	M	49–161	Yes (4 h)	Circulating glucose, serum insulin, HOMA, QUICKI	[↑] Glucose in the MS group vs. Ctl. No differences in insulin and HOMA levels. [↓] QUICKI in the MS group vs. Ctl

Abbreviations: AUC: Area under the curve; CAT: Catalase; Ctl: Control Group; CRFR1: Corticotropin-Releasing Factor type 1 Receptor; F: Females; FRAP: Ferric-Reducing Ability Potential; GPX: Glutathione Peroxidase; GR: Glutathione Reductase; GSH: Reduced Glutathione; GST: Glutathione S Transferase; GTTs: Glucose tolerance Test; HOMA: Homeostasis Model Assessment; HOMA-B: Homeostasis Model Assessment of Beta-cell Function; IL-1β: Interleukin-1β; ISI: Insulin Sensitivity Index; M: Males; MDA: Malondialdehyde; MS: Maternal Separation; NOS: Nitric Oxide Synthase; NR: Not Reported; PND: Postnatal Day; QUICKI: Quantitative Insulin Sensitivity Check Index; SOD: Superoxide Dismutase; ↑: Increase; ↓: Decrease.

**Table 2:** Characteristics of the different maternal separation protocols used in the included studies.

Authors	Beginning of the Separation Period (PND)	Duration of Separation (hours/day)	Length of Separation Period (days)	Conditions of Separation	Weaning (PND)
Schmidt et al. [28]	8	4, 8 or 12	1	In a group, with heating, the mother is in a separate room	NR
George et al. [29]	2	4 (PND 2–5) and 8 (PND 6–16)	15	In a group, with heating, the mother is in a separate room	21 (Ctl), 17 (MS)
Loria et al. [30]	2	3	13	Individual, with heating, mother’s position not specified	28
Maniam & Morris [31]	2	15 min and 3	13	In a group, with heating, the mother is in a separate room	20
Solas et al. [32]	2	3	20	In a group, without further specifications	23
Viveros et al. [33]	9	12 or 24	12 h o 1	In a group, the mother is in the same room	9 or 10
Mela et al. [34]	9	24	1	In a group, the mother is in the same room	22
Paternain et al. [35]	2	3	20	In a group, with heating, the mother is in the same room	23
Bernardi et al. [36]	1	3	10	In a group, with heating, the mother is in a separate room	21
Gehrand et al. [37]	2	1.5	5	In a group, without further specifications	22
Ghosh et al. [38]	2	3	13	In a group, with heating, the mother’s position is not specified.	28
Ho et al. [39]	2	4 (PND 2–5) and 8 (PND 6–16)	15	In a group, without further specifications	21 (Ctl), 17 (MS)
Paternain et al. [40]	2	3	20	In a group, with heating, the mother is in the same room	23
Vargas et al. [41]	1	3	14	Individual, with heating, mother in a separate room	21
Aya-Ramos et al. [42]	1	6	21	In groups, two separations per day, without further specifications	22
Maghami et al. [43]	1	3	21	In a group, with heating, the mother is in a separate room	21
Murphy et al. [44]	2	3	13	Individual, with heating, mother’s position not specified	28
Raff et al. [45]	2	1.5	5	In a group, with heating, the mother’s position is not specified	22
Ruiz et al. [46]	1	3	14	In a group, with heating, the mother’s position is not specified	21
Ilchmann-Diounou et al. [47]	2	3	10	With heating, no further specifications	21
Jaimes-Hoy et al. [48]	2	3	20	In a group, with heating, the mother’s position is not specified	22
Maghami et al. [20]	1	3	21	In a group, with heating, the mother is in a separate room	22
Roque et al. [49]	1	3	14	In a group, with heating, the mother’s position is not specified	21
Eller et al. [50]	1	3	21	In a group, with heating, the mother’s position is not specified	22
Eller et al. [51]	1	3	21	In a group, with heating, the mother’s position is not specified	22
Eskandari et al. [21]	1	3	14	No information is provided	21
Eskandari et al. [22]	1	3	14	Individual, mother in a separate room	21
Fenton-Navarro et al. [52]	1	3	14	Individual, with heating, mother’s position not specified	15
Rico et al. [53]	1	6	21	In a group, with heating, two separations per day, the mother’s position is not specified.	22
Robertson et al. [54]	2	3	16	In a group, with heating, the mother’s position is not specified	21 (Ctl), 17 (MS)

Ctl: Control Group; MS: Maternal Separation; NR: Not Reported; PND: Postnatal Day.

**Table 3:** Risk of bias assessment for included articles (SYRCLEs).

Authors	Sequence Generation	Baseline Characteristics	Allocation Concealment	Random Housing	Blinding of Interventions	Random Outcome Assessment	Blinding of Outcome Assessment	Incomplete Outcome Data	Selective Outcome Reporting	Other Sources of Bias
Schmidt et al. [28]	Unclear	Low	High	Low	High	High	Unclear	Unclear	Unclear	Low
George et al. [29]	Unclear	Low	High	Low	High	High	Unclear	Unclear	Unclear	Low
Loria et al. [30]	High	High	High	Low	High	High	Unclear	High	Unclear	Low
Maniam & Morris [31]	High	Unclear	High	Low	High	High	Unclear	Low	Unclear	Low
Solas et al. [32]	Unclear	Low	High	Low	Unclear	High	Unclear	Unclear	Unclear	Low
Viveros et al. [33]	High	Unclear	High	Low	High	High	Unclear	Unclear	Unclear	Low
Mela et al. [34]	High	Unclear	High	Low	High	High	Unclear	Low	Unclear	Low
Paternain et al. [35]	Low	Low	High	Low	High	High	Unclear	Low	Unclear	Low
Bernardi et al. [36]	High	Unclear	High	Low	High	High	Unclear	Unclear	Unclear	Low
Gehrand et al. [37]	Unclear	Low	High	Low	High	High	Unclear	High	Unclear	Low
Ghosh et al. [38]	Unclear	Low	High	Low	High	High	Unclear	Low	Unclear	Low
Ho et al. [39]	High	Unclear	High	Low	High	High	Unclear	Unclear	Unclear	Low
Paternain et al. [40]	Unclear	Low	High	Low	High	High	Unclear	Low	Unclear	Low
Vargas et al. [41]	Unclear	Low	High	Low	High	High	Unclear	Low	Unclear	Low
Aya-Ramos et al. [42]	Unclear	Low	High	Low	High	High	Unclear	Low	Unclear	Low
Maghami et al. [43]	Unclear	Low	High	Low	High	High	Unclear	Low	Unclear	Low
Murphy et al. [44]	High	Unclear	High	Low	High	High	Unclear	Unclear	Unclear	Low
Raff et al. [45]	High	Unclear	High	Unclear	High	High	Unclear	Low	Unclear	Low
Ruiz et al. [46]	Unclear	Low	High	Low	High	High	Unclear	Unclear	Unclear	Low
Ilchmann-Diounou et al. [47]	High	High	High	Low	High	High	Unclear	Unclear	Unclear	Low
Jaimes-Hoy et al. [48]	Unclear	Low	High	Low	High	High	Unclear	Low	Unclear	Low
Maghami et al. [20]	Unclear	Low	Unclear	Low	High	High	Unclear	Low	Unclear	Low
Roque et al. [49]	High	Unclear	High	Low	High	High	Unclear	Low	Unclear	Low
Eller et al. [50]	High	Unclear	High	Low	High	High	Unclear	Low	Unclear	Low
Eller et al. [51]	High	Unclear	Unclear	Low	High	High	Unclear	Unclear	Unclear	Low
Eskandari et al. [21]	Unclear	Low	High	Unclear	High	High	Unclear	Unclear	Unclear	Low
Eskandari et al. [22]	Unclear	Low	High	Low	High	High	Unclear	Unclear	Unclear	Low
Fenton-Navarro et al. [52]	Unclear	Low	High	Low	High	High	Unclear	Unclear	Unclear	Low
Rico et al. [53]	Unclear	Low	High	Low	High	High	Unclear	Low	Unclear	Low
Robertson et al. [54]	Unclear	Low	Low	Low	Unclear	High	Low	Unclear	Unclear	Low

Regarding random animal housing, the risk was largely low (93%), as this aspect was considered unlikely to affect the results, despite the limited information. Regarding incomplete outcome data, the risk was unclear in almost half of the studies (47%), due to a lack of information on losses or exclusions. Finally, selective outcome reporting was classified as unclear risk in all studies, since no prior protocols were available and no additional relevant sources of bias were found. Therefore, this domain showed a generally low risk.

**Table 4:** Subgroup analysis of the effect of maternal separation on glucose levels.

Glucose								
Subgroups		N Studies	SMD	95% CI	*p*-Value	I^2^ (%)	τ^2^	Subgroup *p*
Species	Rat	15	−0.59	[−0.87; −0.30]	<0.0001	89.2	0.6698	0.0206
	Mice	4	1.09	[−0.30; 2.47]	<0.0001	89.1	3.0760	
Sex	Male	12	−0.77	[−1.15; −0.39]	<0.0001	88.4	0.6588	0.0812
	Female	8	−0.31	[−0.67; 0.05]	<0.0001	86.8	0.4383	
Age	Infant	1	3.38	[2.20; 4.56]	0.8207	0	0	<0.0001
	Adolescent	6	−0.74	[−1.13; −0.34]	<0.0001	90.8	0.6866	
	Young adult	9	−0.14	[−0.48; 0.19]	<0.0001	83.8	0.3099	
	Adult	5	−0.82	[−1.86; 0.21]	<0.0001	91.0	1.2968	
Duration of MS	Moderate	14	−0.65	[−1.11; −0.19]	<0.0001	89.0	0.8466	0.1280
	Severe	5	−0.20	[−0.62; 0.22]	<0.0001	89.6	0.9820	

CI: Confidence Interval; MS: Maternal Separation; SMD: Standardized Mean Difference; Significant differences (*p* < 0.05).

**Table 5:** Subgroup analysis of the effect of maternal separation on insulin levels.

Insulin								
Subgroups		N Studies	SMD	95% CI	*p*-Value	I^2^ (%)	τ^2^	Subgroup *p*
Species	Rat	16	0.01	[−0.32; 0.34]	<0.0001	86.6	0.7771	0.0224
	Mice	2	−0.45	[−0.66; −0.24]	0.3855	0	0.0045	
Sex	Male	11	−0.40	[−0.86; 0.06]	<0.0001	82.7	0.6964	0.0381
	Female	8	0.29	[−0.18; 0.76]	<0.0001	81.8	0.5638	
Age	Adolescent	5	0.12	[−0.57; 0.81]	<0.0001	83.4	0.8611	0.1541
	Young adult	9	0.21	[−0.36; 0.78]	<0.0001	88.4	1.0375	
	Adult	6	−0.44	[−0.88; 0.00]	<0.0001	81.2	0.3760	
Duration of MS	Mild	1	−0.75	[−1.14; −0.36]	0.6184	0	0	<0.0001
	Moderate	17	−0.29	[−0.65; 0.07]	<0.0001	86.8	0.6169	
	Severe	1	1.01	[0.56; 1.45]	0.076	45.5	0.1830	

CI: Confidence Interval; MS: Maternal Separation; SMD: Standardized Mean Difference; Significant differences (*p* < 0.05).

**Table 6:** Subgroup analysis of the effect of maternal separation on the QUICKI index.

QUICKI								
Subgroups		N Studies	SMD	95% CI	*p*-Value	I^2^ (%)	τ^2^	Subgroup *p*
Species	Rat	5	0.89	[0.10; 1.68]	<0.0001	91.4	0.8788	0.3456
	Mice	1	0.37	[−0.36; 1.10]	0.0002	92.7	0.2587	
Sex	Male	1	1.16	[0.75; 1.56]	0.4213	0	0	0.7385
	Female	2	0.73	[−1.75; 3.20]	<0.0001	96.5	3.0787	
Age	Adolescent	2	0.81	[−0.83; 2.44]	<0.0001	95.2	1.3298	0.8469
	Young adult	3	0.97	[−0.16; 2.09]	<0.0001	91.6	0.9037	
	Adult	3	0.52	[−0.53; 1.57]	<0.0001	92.1	0.8068	
Duration of MS	Moderate	6	0.75	[0.14; 1.35]	<0.0001	90.6	0.6877	NA

CI: Confidence Interval; MS: Maternal Separation; NA: Not Applicable; SMD: Standardized Mean Difference; Significant differences (*p* < 0.05).

**Table 7:** Subgroup analysis of the effect of maternal separation on the HOMA index.

HOMA								
Subgroups		N Studies	SMD	95% CI	*p*-Value	I^2^ (%)	τ^2^	Subgroup *p*
Species	Rat	6	0.06	[−0.48; 0.60]	<0.0001	86.7	0.5501	0.2484
	Mice	1	−0.28	[−0.48; −0.07]	0.2644	19.7	0.0043	
Sex	Male	4	−0.10	[−0.84; 0.64]	<0.0001	87.9	0.5082	0.4362
	Female	3	0.45	[−0.72; 1.62]	<0.0001	91.5	1.0070	
Age	Adolescent	3	−0.17	[−0.84; 0.50]	<0.0001	87.5	0.4252	0.7479
	Young adult	2	0.65	[−1.37; 2.68]	<0.0001	96.0	2.0527	
	Adult	2	−0.06	[−0.77; 0.65]	0.0215	81.1	0.2151	
Duration of MS	Moderate	6	−0.03	[−0.77; 0.72]	<0.0001	88.6	0.7987	0.5290
	Severe	1	0.27	[−0.26; 0.79]	0.0260	79.8	0.1138	

CI: Confidence Interval; MS: Maternal Separation; SMD: Standardized Mean Difference; Significant differences (*p* < 0.05).

**Table 8:** Subgroup analysis of the effect of maternal separation on GTTs.

Glucose Tolerance Test								
Subgroups		N Studies	SMD	95% CI	*p*-Value	I^2^ (%)	τ^2^	Subgroup *p*
Species	Rat	4	−1.01	[−1.22; −0.80]	0.4867	0	0	0.4632
	Mice	1	−1.49	[−2.74; −0.24]	–	NA	NA	
Sex	Male	4	−1.14	[−1.40; −0.87]	0.7522	0	0	0.0977
	Female	1	−0.72	[−1.14; −0.29]	–	NA	NA	
Age	Adolescent	1	−1.08	[−1.64; −0.51]	–	NA	NA	0.5634
	Young adult	2	−0.96	[−1.23; −0.69]	0.2683	23.9	0.0085	
	Adult	2	−1.28	[−1.81; −0.75]	0.7262	0	0	
Duration of MS	Moderate	5	−1.02	[−1.23; −0.82]	0.5522	0	0	NA

CI: Confidence Interval; MS: Maternal Separation; NA: Not Applicable; SMD: Standardized Mean Difference; Significant differences (*p* < 0.05).

## Data Availability

The original contributions presented in this study are included in the article/Supplementary Materials. Further inquiries can be directed to the corresponding author.

## Supplementary Materials

The following supporting information can be downloaded at: https://www.mdpi.com/article/10.3390/ijms26209927/s1.

## Abbreviations

The following abbreviations are used in this manuscript:

ACEs	Adverse childhood experiences
CAT	Catalase
FRAP	Ferric-Reducing Ability Potential
GPx	Glutathione peroxidase
GR	Glutathione reductase
GSH	Reduced glutathione
GST	Glutathione S-transferase
GTTs	Glucose tolerance tests
HOMA	Homeostasis model assessment
HOMA-B	Homeostasis model assessment of Beta-cell function
HPA	Hypothalamic–pituitary–adrenal
IGF-1	Insulin-like growth factor 1
IL-1β	Interleukin-1β
ISI	Insulin sensitivity index
ITTs	Insulin tolerance tests
MDA	Malondialdehyde
MS	Maternal separation
NOS	Nitric oxide synthase
PND	Postnatal day
QUICKI	Quantitative insulin sensitivity check index
SOD	Superoxide dismutase
