# Supplementary material for: Impact of Early Postnatal Maternal Separation Stress on Pancreatic Function in Rodents: A Systematic Review and Meta-Analysis
Source: Int J Mol Sci. 2025 Oct 12;26(20):9927. doi: 10.3390/ijms26209927 (PMC12564252; doi:10.3390/ijms26209927)
Supplement: Supplementary file 1 [file ijms-26-09927-s001.zip › ijms-3904837-supplementary.pdf]

SUPPLEMENTARY INFORMATION (SI) for

Impact of Early Postnatal Maternal Separation Stress on Pancreatic Morphology and Function in Rodent: A Systematic Review and Meta-analysis.

SI 1

Table S1. Search strategies and results obtained for each information source used (N = 491).

Sources	Search strategies
**PubMed (n = 74)**	("Rodentia"[MeSH Terms] OR "Mice"[MeSH Terms] OR "Rats"[MeSH Terms] OR "rodentia*"[Title/Abstract] OR "Mice"[Title/Abstract] OR "Rats"[Title/Abstract] OR "Rat"[Title/Abstract] OR "rodent*"[Title/Abstract] OR "Rattus"[Title/Abstract] OR "Mus"[Title/Abstract] OR "Mouse"[Title/Abstract] OR "Murine"[Title/Abstract] OR "Mus musculus"[Title/Abstract] OR (("Rat"[Title/Abstract] OR "Rats"[Title/Abstract] OR "Mouse"[Title/Abstract]) AND "Laboratory"[Title/Abstract])) AND ("Maternal Deprivation"[MeSH Terms] OR "maternal deprivation*"[Title/Abstract] OR "Deprivation maternal"[Title/Abstract] OR "Maternal separation"[Title/Abstract] OR "neonatal stress*"[Title/Abstract] OR "postnatal stress*"[Title/Abstract] OR "early life stress*"[Title/Abstract] OR "Early Life Stresses"[Title/Abstract] OR "Life Stress Early"[Title/Abstract] OR "Stress Early Life"[Title/Abstract]) AND ("Pancreas"[MeSH Terms] OR "Insulin"[MeSH Terms] OR "Pancreatic Polypeptide"[MeSH Terms] OR "Pancreas"[Title/Abstract] OR "Islets of Langerhans"[Title/Abstract] OR "Langerhans Islets"[Title/Abstract] OR "islet cell*"[Title/Abstract] OR "cell* islet"[Title/Abstract] OR "pancreatic islet*"[Title/Abstract] OR "islet* pancreatic"[Title/Abstract] OR "Islands of Langerhans"[Title/Abstract] OR "Langerhans Islands"[Title/Abstract] OR "glucagon secreting cell*"[Title/Abstract] OR "pancreatic alpha cell*"[Title/Abstract] OR "pancreatic a cell*"[Title/Abstract] OR "alpha cell* pancreatic"[Title/Abstract] OR "insulin secreting cell*"[Title/Abstract] OR "pancreatic beta cell*"[Title/Abstract] OR "beta cell* pancreatic"[Title/Abstract] OR "pancreatic b cell*"[Title/Abstract] OR "Insulin"[Title/Abstract] OR "pancreatic polypeptide cell*"[Title/Abstract] OR "somatostatin secreting cell*"[Title/Abstract] OR "d cell*"[Title/Abstract] OR "somatostatin cell*"[Title/Abstract] OR "delta cell*"[Title/Abstract])
**Scopus (n = 114)**	TITLE-ABS-KEY (("Rodentia*" OR "Mice" OR "Rats" OR "Rat" OR "Rodent*" OR "Rattus" OR "Mus" OR "Murine" OR "Laboratory Rat*") AND ("Maternal deprivation*" OR "Deprivation maternal" OR "Maternal separation" OR "Neonatal stress*" OR "Postnatal stress*" OR "Early Life Stress*" OR "Life Stress Early" OR "Stress Early Life") AND ("Pancreas" OR "Islets of Langerhans" OR "Langerhans Islets" OR "Islet Cell*" OR "Cell*, Islet" OR "Pancreatic Islet*" OR "Islet* Pancreatic" OR "Islands of Langerhans" OR "Langerhans Islands" OR "Glucagon Secreting-Cell*" OR "Pancreatic alpha Cell*" OR "Pancreatic A Cell*" OR "alpha Cell*, Pancreatic" OR "Insulin-Secreting Cell*" OR "Pancreatic beta Cell*" OR "beta Cell*, Pancreatic" OR "Pancreatic B Cell*" OR "Insulin" OR "Pancreatic Polypeptide Cell*" OR "Pancreatic Polypeptide" OR "Somatostatin-Secreting Cell*" OR "D Cell*" OR "Somatostatin Cell*" OR "delta Cell*"))
**EMBASE (n = 105)**	('rodent'/exp OR 'rodent' OR 'rodentia':ab,ti OR 'mice':ab,ti OR 'rat':ab,ti OR 'rats':ab,ti OR 'rodent*':ab,ti OR 'rattus':ab,ti OR 'mus':ab,ti OR 'mouse':ab,ti OR 'murine':ab,ti) AND ('maternal deprivation'/exp OR 'maternal deprivation*':ab,ti OR 'maternal separation':ab,ti OR 'deprivation maternal':ab,ti OR 'neonatal stress*':ab,ti OR 'postnatal stress*':ab,ti OR 'early life stress*':ab,ti OR 'life stress early':ab,ti OR 'stress early life':ab,ti) AND ('pancreas'/exp OR 'pancreas':ab,ti OR 'islets of langerhans':ab,ti OR 'langerhans islets':ab,ti OR 'islet cell*':ab,ti OR 'cell*, islet':ab,ti OR 'pancreatic islet*':ab,ti OR 'islet* pancreatic':ab,ti OR 'islands of langerhans':ab,ti OR 'langerhans islands':ab,ti OR 'glucagon secreting-cell*':ab,ti OR 'pancreatic alpha cell*':ab,ti OR 'pancreatic a cell*':ab,ti OR 'alpha cell*, pancreatic':ab,ti OR 'insulin-secreting cell*':ab,ti OR 'pancreatic beta cell*':ab,ti OR 'beta cell*, pancreatic':ab,ti OR 'pancreatic b cell*':ab,ti OR 'insulin':ab,ti OR 'insulin'/exp OR 'pancreatic

	polypeptide cell*':ab,ti OR 'pancreas polypeptide'/exp OR 'pancreatic polypeptide':ab,ti OR 'somatostatin-secreting cell*':ab,ti OR 'd cell*':ab,ti OR 'somatostatin cell*':ab,ti OR 'delta cell*':ab,ti)
**WoS (n = 115)**	TS=(("Rodentia*" OR "Mice" OR "Rats" OR "Rat" OR "Rodent*" OR "Rattus" OR "Mus" OR "Mouse" OR "Murine" OR "Mus musculus") AND ("Maternal deprivation*" OR "Deprivation maternal" OR "Maternal separation" OR "Neonatal stress*" OR "Postnatal stress*" OR "Early Life Stress*" OR "Life Stress Early" OR "Stress Early Life") AND ("Pancreas" OR "Islets of Langerhans" OR "Langerhans Islets" OR "Islet Cell*" OR "Cell*, Islet" OR "Pancreatic Islet*" OR "Islet* Pancreatic" OR "Islands of Langerhans" OR "Langerhans Islands" OR "Glucagon Secreting-Cell*" OR "Pancreatic alpha Cell*" OR "Pancreatic A Cell*" OR "alpha Cell*, Pancreatic" OR "Insulin-Secreting Cell*" OR "Pancreatic beta Cell*" OR "beta Cell*, Pancreatic" OR "Pancreatic B Cell*" OR "Insulin" OR "Pancreatic Polypeptide Cell*" OR "Pancreatic Polypeptide" OR "Somatostatin-Secreting Cell*" OR "D Cell*" OR "Somatostatin Cell*" OR "delta Cell*"))
**BIREME - BVS (n = 83)**	(("Rodentia*" OR "Mice" OR "Rats" OR "Rat" OR "Rodent*" OR "Rattus" OR "Mus" OR "Mouse" OR "Murine" OR "Roedores" OR "Ratones" OR "Ratón" OR "Rata" OR "Ratas" OR "Murinae" OR "Camundongos" OR "Camundongo" OR "Ratos" OR "Rato")) AND (("Maternal deprivation*" OR "Deprivation maternal" OR "Maternal separation" OR "Neonatal stress*" OR "Postnatal stress*" OR "Early Life Stress*" OR "Life Stress Early" OR "Stress Early Life" OR "Privación Materna" OR "Separación Materna" OR "Privação Materna")) AND (("Pancreas" OR "Islets of Langerhans" OR "Langerhans Islets" OR "Islet Cell*" OR "Cell*, Islet" OR "Pancreatic Islet*" OR "Islet* Pancreatic" OR "Islands of Langerhans" OR "Langerhans Islands" OR "Glucagon Secreting-Cell*" OR "Pancreatic alpha Cell*" OR "Pancreatic A Cell*" OR "alpha Cell*, Pancreatic" OR "Insulin-Secreting Cell*" OR "Pancreatic beta Cell*" OR "beta Cell*, Pancreatic" OR "Pancreatic B Cell*" OR "Insulin" OR "Pancreatic Polypeptide" OR "Somatostatin-Secreting Cell*" OR "D Cell*" OR "Somatostatin Cell*" OR "delta Cell*" OR "Células de los Islotes Pancreáticos" OR "Células beta Pancreáticas" OR "Ilhotas Pancreáticas"))
**SciELO (n = 0)**	(("Rodentia*" OR "Mice" OR "Rats" OR "Rat" OR "Rodent*" OR "Rattus" OR "Mus" OR "Mouse" OR "Murine" OR "Roedores" OR "Ratones" OR "Ratón" OR "Rata" OR "Ratas" OR "Murinae" OR "Camundongos" OR "Camundongo" OR "Ratos" OR "Rato")) AND (("Maternal deprivation*" OR "Deprivation maternal" OR "Maternal separation" OR "Neonatal stress*" OR "Postnatal stress*" OR "Early Life Stress*" OR "Life Stress Early" OR "Stress Early Life" OR "Privación Materna" OR "Separación Materna" OR "Privação Materna")) AND (("Pancreas" OR "Islets of Langerhans" OR "Langerhans Islets" OR "Islet Cell*" OR "Cell*, Islet" OR "Pancreatic Islet*" OR "Islet* Pancreatic" OR "Islands of Langerhans" OR "Langerhans Islands" OR "Glucagon Secreting-Cell*" OR "Pancreatic alpha Cell*" OR "Pancreatic A Cell*" OR "alpha Cell*, Pancreatic" OR "Insulin-Secreting Cell*" OR "Pancreatic beta Cell*" OR "beta Cell*, Pancreatic" OR "Pancreatic B Cell*" OR "Insulin" OR "Pancreatic Polypeptide" OR "Somatostatin-Secreting Cell*" OR "D Cell*" OR "Somatostatin Cell*" OR "delta Cell*" OR "Células de los Islotes Pancreáticos" OR "Células beta Pancreáticas" OR "Ilhotas Pancreáticas"))

SI 2.1

Species

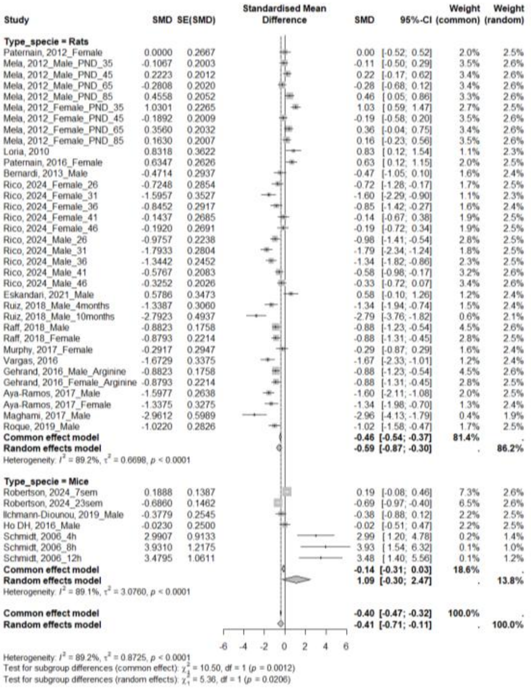

Sex

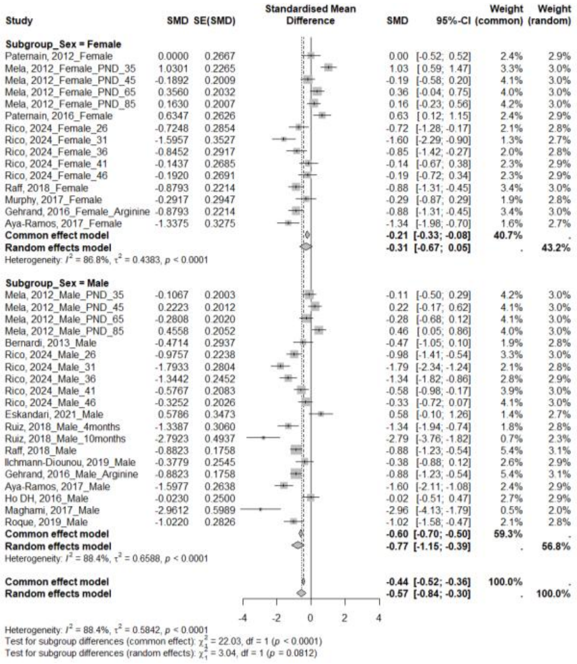

Age at the time of the test

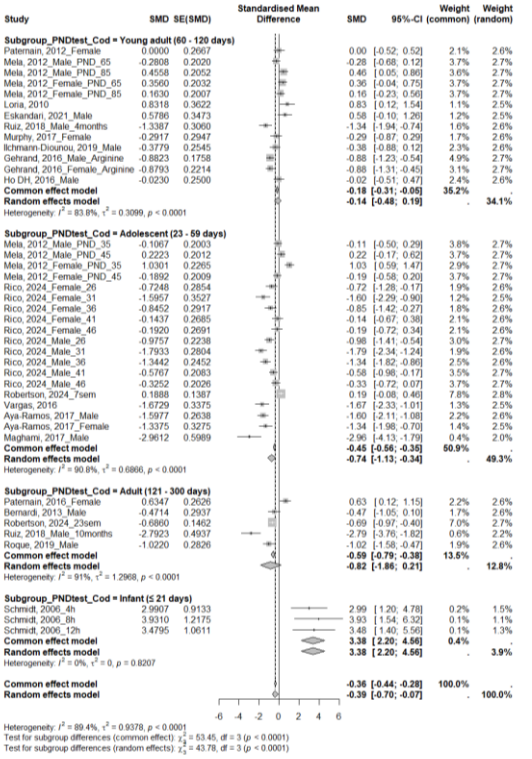

Duration of MS

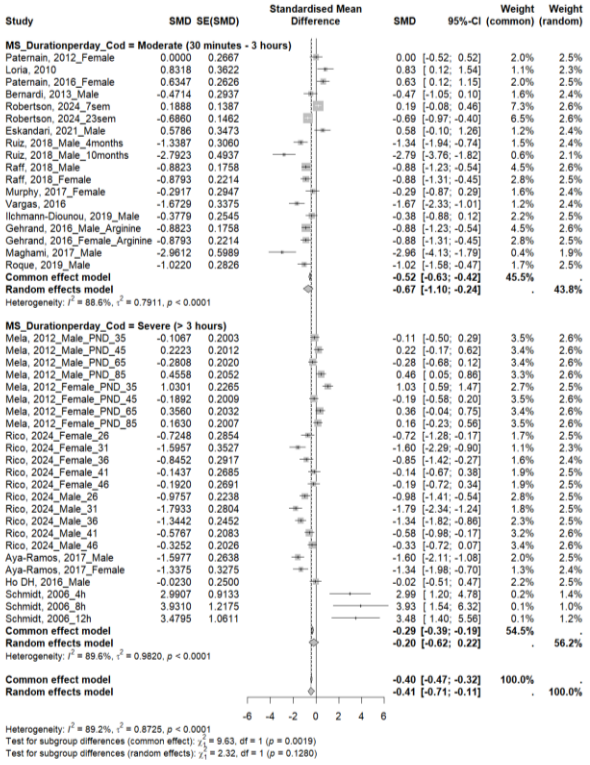

Figure S1. Forest plots of subgroup meta-analysis on the effect of MS on glucose levels.

SI 2.2

Species

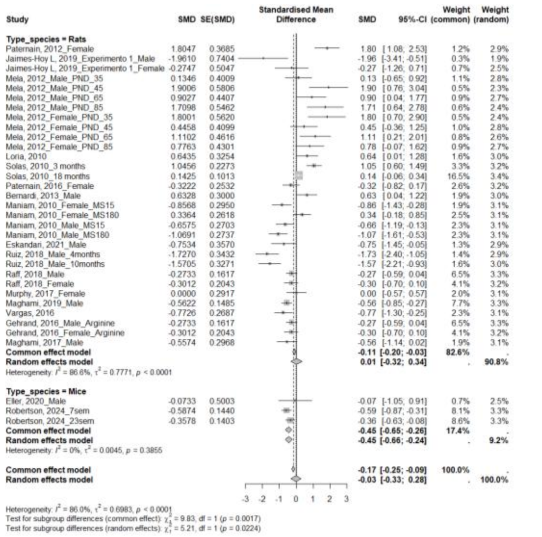

Sex

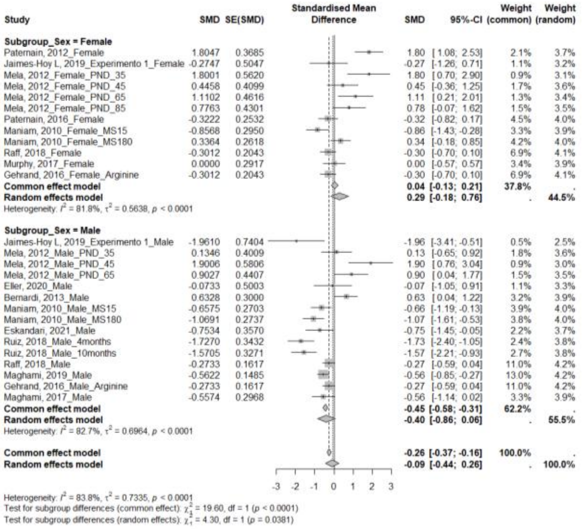

Age at the time of the test

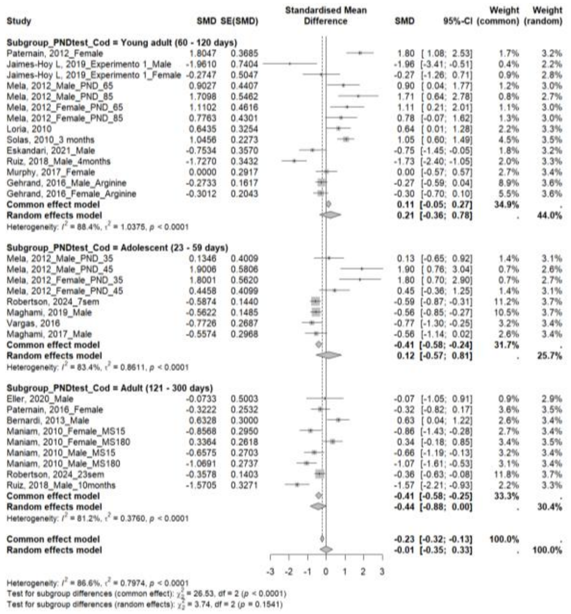

Duration of MS

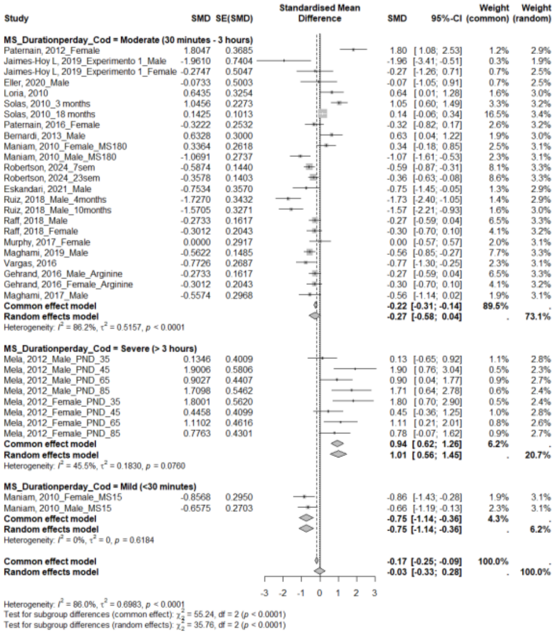

Figure S2. Forest plots of subgroup meta-analysis on the effect of MS on insulin levels.

SI 2.3

Species

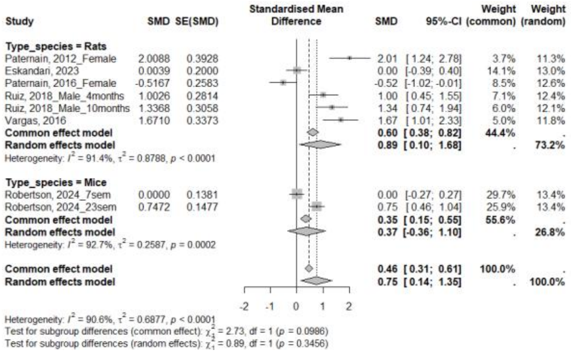

Sex

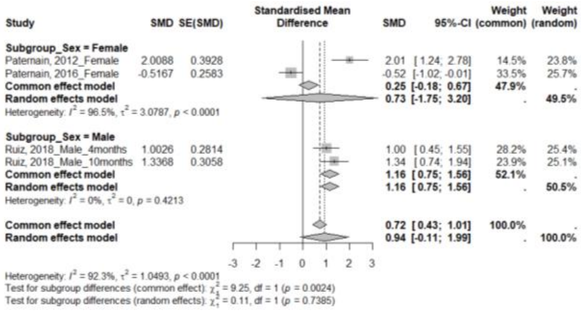

Age at the time of the test

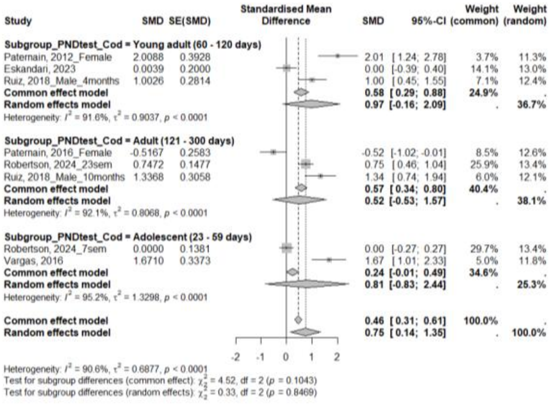

Duration of MS

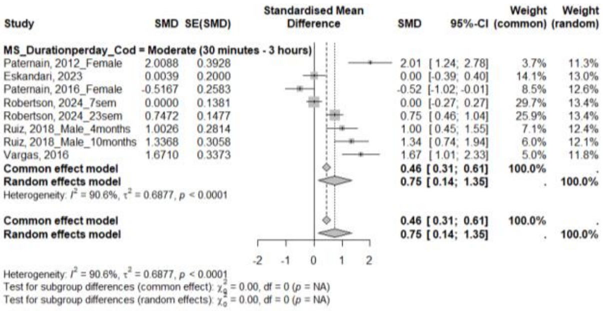

Figure S3. Forest plots of the subgroup meta-analysis on the effect of MS on the QUICKI index.

SI 2.4

Species

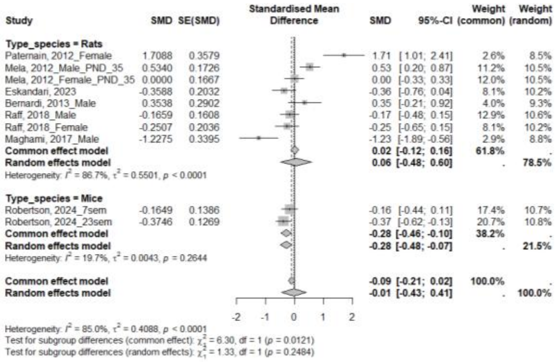

Sex

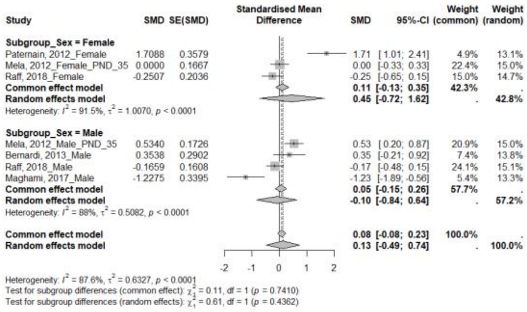

Age at the time of the test

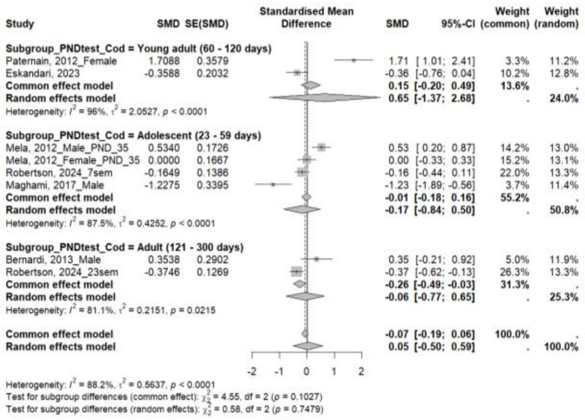

Duration of MS

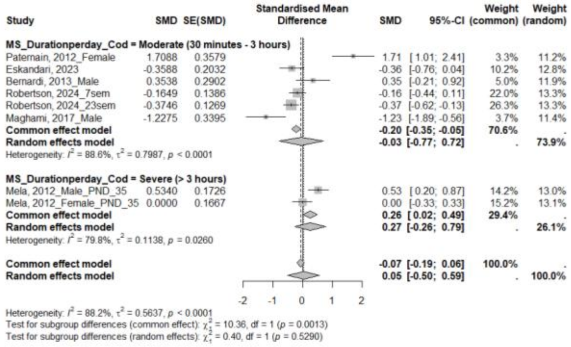

Figure S4. Forest plots of the subgroup meta-analysis on the effect of MS on the HOMA index.

SI 2.5

Species

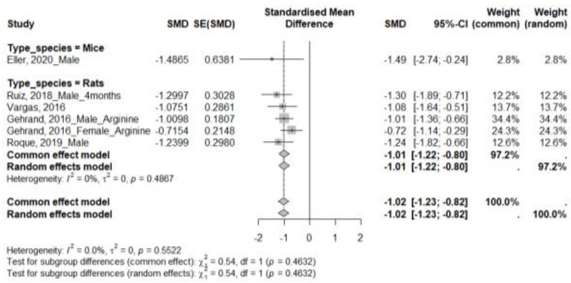

Sex

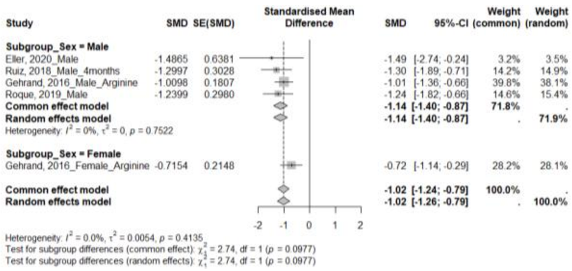

Age at the time of the test

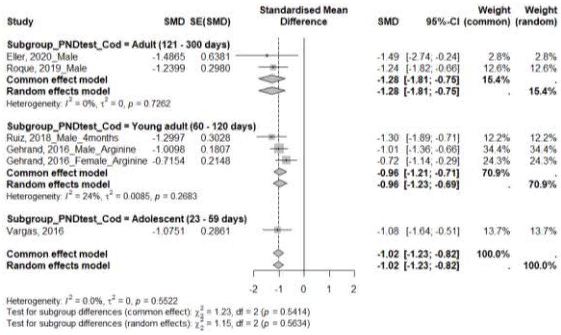

Duration of MS

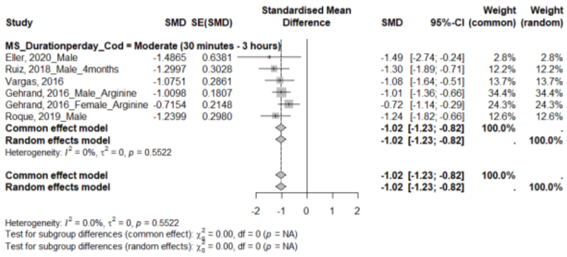

Figure S5. Forest plots of the subgroup meta-analysis on the effect of MS on the glucose tolerance test.
